# Oral Supplementation With Butyrate Improves Myocardial Ischemia/Reperfusion Injury *via* a Gut-Brain Neural Circuit

**DOI:** 10.3389/fcvm.2021.718674

**Published:** 2021-09-23

**Authors:** Zhiyao Yu, Jiapeng Han, Huaqiang Chen, Yueyi Wang, Liping Zhou, Meng Wang, Rong Zhang, Xiaoxing Jin, Guocheng Zhang, Changyi Wang, Tianyou Xu, Mengjie Xie, Xiaofei Wang, Xiaoya Zhou, Hong Jiang

**Affiliations:** ^1^Department of Cardiology, Renmin Hospital, Wuhan University, Wuhan, China; ^2^Cardiac Autonomic Nervous System Research Center, Wuhan University, Wuhan, China; ^3^Cardiovascular Research Institute, Wuhan University, Wuhan, China; ^4^Hubei Key Laboratory of Cardiology, Wuhan, China

**Keywords:** short-chain fatty acid, butyrate, myocardial ischemia reperfusion injury, gut-brain axis, sympathetic nervous system

## Abstract

**Objective:** Butyrate, a short-chain fatty acid (SCFA) produced by the intestinal microbiota, plays a protective role in cardiovascular diseases (CVDs), but the mechanisms involved in this process remain unelucidated. We aimed to explore the effect of butyrate on myocardial ischemia/reperfusion (I/R) injury through the gut-brain neural circuit.

**Methods:** Rats were randomly divided into four groups: sham group (sham), I/R group (I/R), I/R+ butyrate group (butyrate), and I/R+ butyrate+ vagotomy group (vagotomy). The rats were treated with sodium butyrate for 4 weeks, and the gut-brain neural circuit was investigated by subdiaphragmatic vagotomy.

**Results:** Butyrate treatment significantly reduced the infarct size and decreased the expression of creatine kinase (CK), creatine kinase myocardial isoenzyme (CK-MB), and lactate dehydrogenase (LDH) compared with the values found for the I/R group. In addition, the I/R-induced increases in inflammation, oxidative stress, and apoptosis were attenuated by butyrate. However, the above-mentioned protective effects were diminished by subdiaphragmatic vagotomy. The RNA sequencing results also revealed that the butyrate-induced protective changes at the cardiac transcription level were reversed by vagotomy. An analysis of the heart rate variability (HRV) and the detection of norepinephrine (NE) showed that butyrate significantly inhibited the I/R-induced autonomic imbalance, but this inhibition was not observed in the vagotomy group. Butyrate treatment also suppressed the neural activity of the paraventricular nucleus (PVN) and superior cervical ganglion (SCG), and both of these effects were lost after vagotomy.

**Conclusions:** Butyrate treatment significantly improves myocardial I/R injury via a gut-brain neural circuit, and this cardioprotective effect is likely mediated by suppression of the sympathetic nervous system.

## Introduction

Acute myocardial infarction (AMI) is one of the leading causes of death worldwide. Reperfusion is a required event that induces a further process called myocardial ischemia/reperfusion (I/R) injury, which includes inflammation, oxidative stress, and apoptosis, and induces cardiomyocyte damage (1–3). However, the prevention and treatment of myocardial I/R injury remain limited.

The gut microbiota plays an important role in cardiovascular health. Bacterial metabolites can mediate interactions with distant organs. Short-chain fatty acids (SCFAs) constitute a major class of metabolites that are mainly produced in the colon by bacterial fermentation (4). The concentration of SCFAs could be regulated by the gut microbiota and diet. Both a low-fiber diet and a decrease in SCFA-producing bacteria could exert harmful effects on cardiovascular diseases (CVDs) (5, 6). Butyrate is one of the predominant SCFAs, and supplementation has been shown to be protective against myocardial infarction, hypertension, and other diseases (7, 8). The mechanisms mainly focus on the activation of G-coupled receptors (GPRs) or the inhibition of histone deacetylases (HDACs). However, in addition to its direct effects on cardiomyocytes, accumulating evidence suggests that oral supplementation with butyrate may induce effects via gut-brain neural mechanisms, which depend on afferent vagus nerve signaling (9–11). The gut-brain axis is a bidirectional pathway connecting the central nervous system and the gastrointestinal tract and is associated with multiple diseases. The vagus nerve is a vital connection that links the gut and brain (12). Therefore, we hypothesized that butyrate might improve myocardial I/R injury via a gut-brain neural circuit.

Moreover, the pathogenesis of myocardial ischemia is complex and involves an autonomic imbalance between sympathetic system hyperactivity and parasympathetic system hypoactivity (13, 14). The paraventricular nucleus (PVN) is an important central sympathetic nucleus that plays a critical role in regulating sympathetic excitation and cardiovascular function (15). Anatomical and functional evidence shows that neural pathways from the gut to the PVN are biologically plausible (16). Therefore, we aimed to evaluate the effect of butyrate on myocardial I/R and hypothesized that the contribution of the gut-brain neural circuit may be in part due to suppression of the sympathetic nervous system.

## Materials and Methods

### Animals

Healthy male Sprague-Dawley rats (200–250 g) were used in this study and maintained in accordance with the Guidelines for the Care and Use of Laboratory Animals (US National Institutes of Health). All experiments were approved by the Animal Welfare & Ethics Committee of Renmin Hospital of Wuhan University. All animals were maintained under an alternating 12-h dark/12-h light cycle in a humidity/temperature-controlled room (70% relative humidity and 23°C) and had free access to food and water.

### Animal Model and Dietary Intervention

**Figure 1:**
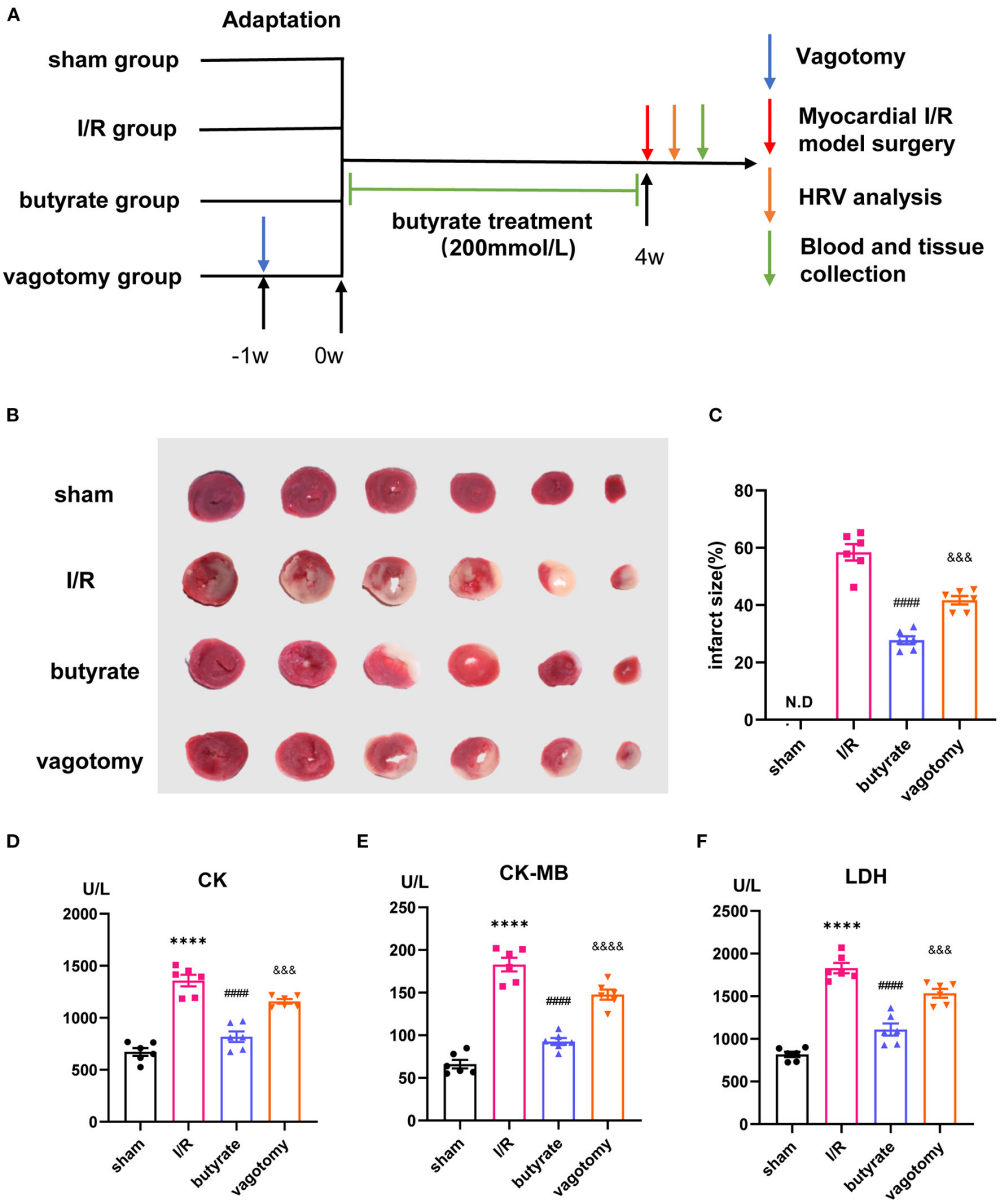
Butyrate treatment attenuated myocardial damage after myocardial I/R, and vagotomy diminished this protective effect. **(A)** Timeline of vagotomy, myocardial I/R model surgery, and butyrate treatment. **(B)** Representative images of heart sections by TTC staining. **(C)** Quantitative analysis of infarct size expressed as percentage of left ventricle, N.D. no detectable. **(D)** Serum CK, **(E)** serum CK-MB, and **(F)** serum LDH levels. ****p < 0.0001 vs. sham group; ^####^*p* < 0.0001 vs. I/R group; ^&&&^*p* < 0.001, ^&&&&^*p* < 0.0001 vs. butyrate group. I/R, ischemia/reperfusion; HRV, heart rate variability; CK, creatine kinase; CK-MB, creatine kinase-MB; LDH, lactate dehydrogenase.

The rats were randomly divided into four groups (*n* = 6 each): (1) sham group, (2) I/R group, (3) I/R +butyrate group (butyrate group), and (4) I/R +butyrate +vagotomy group (vagotomy group). To study the effects of butyrate, the rats received sodium butyrate (200 mmol/L, Sigma-Aldrich) in drinking water *ad libitum* for 4 weeks. The rats in the vagotomy group were subjected to subdiaphragmatic vagotomy surgery as previously described (17). After 1 week of recovery from the surgery, the rats received supplementation with sodium butyrate. After 4 weeks of sodium butyrate supplementation, the rats were anesthetized by injection with 2% pentobarbital sodium (40 mg/kg body weight, i.p.). After a median sternotomy, the proximal left anterior descending coronary artery was ligated with 6-0 silk sutures. The rats in the I/R, butyrate, and vagotomy groups were subjected to ischaemia for 45 min and reperfusion for 24 h as previously described (18). The rats in the sham group underwent sham surgery (Figure 1A).

### Subdiaphragmatic Vagotomy

Rats were anesthetized by injection with 2% pentobarbital sodium (40 mg/kg body weight, i.p.), and a midline abdominal incision was then made along the linea alba. The liver was carefully moved to expose the esophagus. The right and left vagus nerves were visualized along the esophagus below the diaphragm and cut. According to the previous studies, anatomical verification of subdiaphragmatic vagotomy was conducted by successfully dissecting and visualizing the vagal nerves below the diaphragm and enlarged stomachs (11). Taking into consideration that the observed effects in vagotomy group may be due to vagotomy itself and irrelevant to diminished effects of butyrate, we performed preliminary experiment to evaluate the effect of subdiaphragmatic vagotomy on myocardial I/R. We measured the CK-MB, SOD, IL-6, and HRV levels in I/R group and I/R+vagotomy group. The results suggested no statistical difference between these two groups (Supplementary Figure 1), indicated that vagotomy, itself, did not have a significant effect on myocardial I/R.

### Measurement of the Heart Rate Variability

A 15-min electrocardiogram was recorded to measure the heart rate variability (HRV) using a PowerLab data acquisition system (AD Instruments, New South Wales, Australia). Frequency domain parameters, namely, low frequency (LF), high frequency (HF), and the low-frequency/high-frequency ratio (LF/HF ratio), were determined as described previously (19).

### Triphenyl Tetrazolium Chloride Staining

The hearts were frozen rapidly and chopped into six 1-mm-thick sections. The sections were incubated at 37°C with 1% triphenyl tetrazolium chloride (TTC) in phosphate buffer (pH 7.4) for 10 min and fixed in 10% formaldehyde solution. The areas of ischaemic and non-ischaemic left ventricles (LVs) were calculated using Image-Pro Plus v.6.0 (Media Cybernetics, Silver Spring, MD, USA). The infarct size was expressed as a percentage of the infarct volume vs. the LV volume.

### Serum Biochemistry and ELISA

Blood samples were collected 24 h after reperfusion, and plasma was isolated according to a standard separation procedure involving centrifugation at 3,000 rpm and 4°C for 15 min and stored at −80°C. The serum levels of creatine kinase (CK), creatine kinase-myocardial isoenzyme (CK-MB), and lactate dehydrogenase (LDH) were analyzed using commercial kits (Nanjing Jiancheng Bioengineering Institute, Nanjing, China) following the manufacturer's protocols. The serum norepinephrine (NE) (Cusabio Company, Wuhan, China), IL-6 (R&D Systems, MN, USA), IL-1β (MULTISCIENCES BIOTECH, Hangzhou, China), and TNF-α (Thermo Fisher Scientific, MA, USA) levels were measured by ELISA according to the manufacturer's recommendations.

### Determination of the Tissue Malondialdehyde Level and Superoxide Dismutase Activity

Aliquots of tissue samples from the LV were completely homogenized. The malondialdehyde (MDA) levels and superoxide dismutase (SOD) activity were measured using chemical assay kits (Nanjing Jiancheng Bioengineering Institute).

### Real-Time PCR Analysis

Aliquots of tissue samples from the LV were completely homogenized. The mRNA expression levels of IL-6 and TNF-α were detected by RT-qPCR. Total RNA was extracted from LV tissues using the TRIzol reagent (Invitrogen™, Thermo) following the manufacturer's instructions. The RNA concentration was measured using a NanoDrop2000 (Thermo). Subsequently, cDNA was synthesized using a Revert Aid First-Strand cDNA Synthesis Kit (Thermo). GAPDH was used for normalization. The gene expression levels were determined using the 2-ΔΔCT method. The primer sequences were as follows: IL-6 forward, AGGATACCACCCACAACAGACC, and reverse, TTGCCATTGCACAACTCTTTTC; TNF-α forward, CCAGGTTCTCTTCAAGGGACAA, and reverse, GGTATGAAATGGCAAATCGGCT; and GAPDH forward, CTGGAGAAACCTGCCAAGTATG, and reverse, GGTGGAAGAATGGGAGTTGCT.

### Western Blotting

Snap-frozen LV tissue of the heart was obtained and lysed with RIPA solution (Beyotime Institute). Isolated protein (40 μg) was separated by 10% SDS-PAGE, transferred onto polyvinylidene fluoride membranes (PVDF, Millipore, USA), and incubated at room temperature for 1 h. The membranes were then incubated with primary antibody overnight at 4°C. The following primary antibodies were used: Bax (1:1,000, Cell Signaling Technology, Boston, MA, USA) and Bcl-2 (1:1,000, R&D Systems, MN, USA). After four 5-min washes in TBST, the membranes were incubated with HRP-goat anti-rabbit (1:3,000, Elabscience, Wuhan, China) and HRP-goat anti-mouse (1:3,000, Elabscience, Wuhan, China) antibodies at room temperature for 1 h in the dark. After washes with TBST, the band intensities were analyzed using the Odyssey Imaging System (LICOR Biosciences, Lincoln, NE, USA).

### TUNEL Staining Analysis

Apoptosis in myocardial tissue was determined with the TUNEL kit (Roche, Germany) according to the manufacturer's instructions. The positive granules were observed by fluorescence microscopy and quantified by Image-Pro Plus v.6.0 (Media Cybernetics, Silver Spring, MD, USA) using five fields per section in each slide in a blinded manner.

### Immunofluorescence Analysis

Brain and superior cervical ganglion (SCG) tissues were rapidly collected and fixed in 4% paraformaldehyde. The borders of the PVN were determined based on a rat brain atlas, and 5-μm sections were used for immunofluorescence analysis. Antibodies against cFos (Servicebio, Wuhan, China) and tyrosine hydroxylase (TH) (Servicebio, Wuhan, China) were used. The expression of cFos in the SCG and PVN was quantified to evaluate neuronal activity. All quantitative analyses were performed with Image-Pro Plus (Media Cybernetics, Inc., Rockville, MD, USA).

### RNA Sequencing

For RNA sequencing (RNA-Seq) studies, the ischemic myocardium was rapidly excised and snap-frozen in liquid nitrogen for storage. Total RNA was extracted from the tissues using TRIzol (Invitrogen, Carlsbad, CA, USA) according to the instructions provided in the manual and then qualified and quantified using a NanoDrop and Agilent 2100 bioanalyser (Thermo Fisher Scientific, MA, USA). Oligo(dT)-attached magnetic beads were used for the purification of mRNA, and single-strand circle DNA (ssCir DNA) was then formatted as the final library. The BGSEQ-500 (BGI-Shenzhen, China) platform was used for gene sequencing to obtain raw reads. The clean reads were mapped to the rat genome (Rattus_norvegicus, UCSC, rn6) using HISAT2 (Hierarchical Indexing for Spliced Alignment of Transcripts, v2.0.4), and Bowtie2 (v2.2.5; -q –phred64 –sensitive –dpad 0 –gbar 99999999 –mp 1,1 –np 1 –score-min L,0, 0.1 -p 16 -k 200) was utilized to align the clean reads to the reference coding gene set. RSEM (version 1.2.12; default) was then used to calculate the expression level, and DEGseq2 (v1.4.5) was used with the following predefined parameters: “Fold Change ≥2 and Adjusted *P*-value (*Q*-value) ≤0.05.” The heatmap function of the R software package was applied to depict these differentially expressed genes in a heatmap. The gene ontology (GO) enrichment and Kyoto Encyclopedia of Genes and Genomes (KEGG) pathway classification results were analyzed using the R software package. The *P*-value was adjusted by FDR, and the adjusted *P*-value was designated the *Q*-value. The significance level was set to *Q*-value ≤ 0.05.

### Statistical Analysis

All the data are presented as the means ± standard errors of the mean (SEMs). Differences were compared by one-way analysis of variance (ANOVA) followed by Tukey's multiple comparison *post hoc test*. All statistical analyses were performed using GraphPad Prism 9 software. *P-*values < 0.05 were considered statistically significant.

## Results

### Butyrate Treatment Attenuated Myocardial Damage After Myocardial I/R, and Vagotomy Diminished This Protective Effect

We investigated whether butyrate was protective against myocardial I/R injury. An examination of the infarct size showed that the butyrate group presented a significantly lower infarct size than the I/R group. Moreover, the rats in the vagotomy group had a significantly higher infarct size than those in the butyrate group (Figures 1B,C).

In addition, we measured the plasma LDH, CK, and CK-MB levels as indicators of myocardial damage. Higher levels of LDH, CK, and CK-MB leakage were observed in the I/R group, and these increases were significantly attenuated by butyrate treatment. In contrast, vagotomy significantly increased the release of LDH, CK, and CK-MB (Figures 1D–F). These findings indicate that butyrate treatment decreases myocardial damage after I/R and that vagotomy diminishes this protective effect.

### Butyrate Treatment Improved Oxidative Stress and Cell Apoptosis After Myocardial I/R, and Vagotomy Diminished This Protective Effect

**Figure 2:**
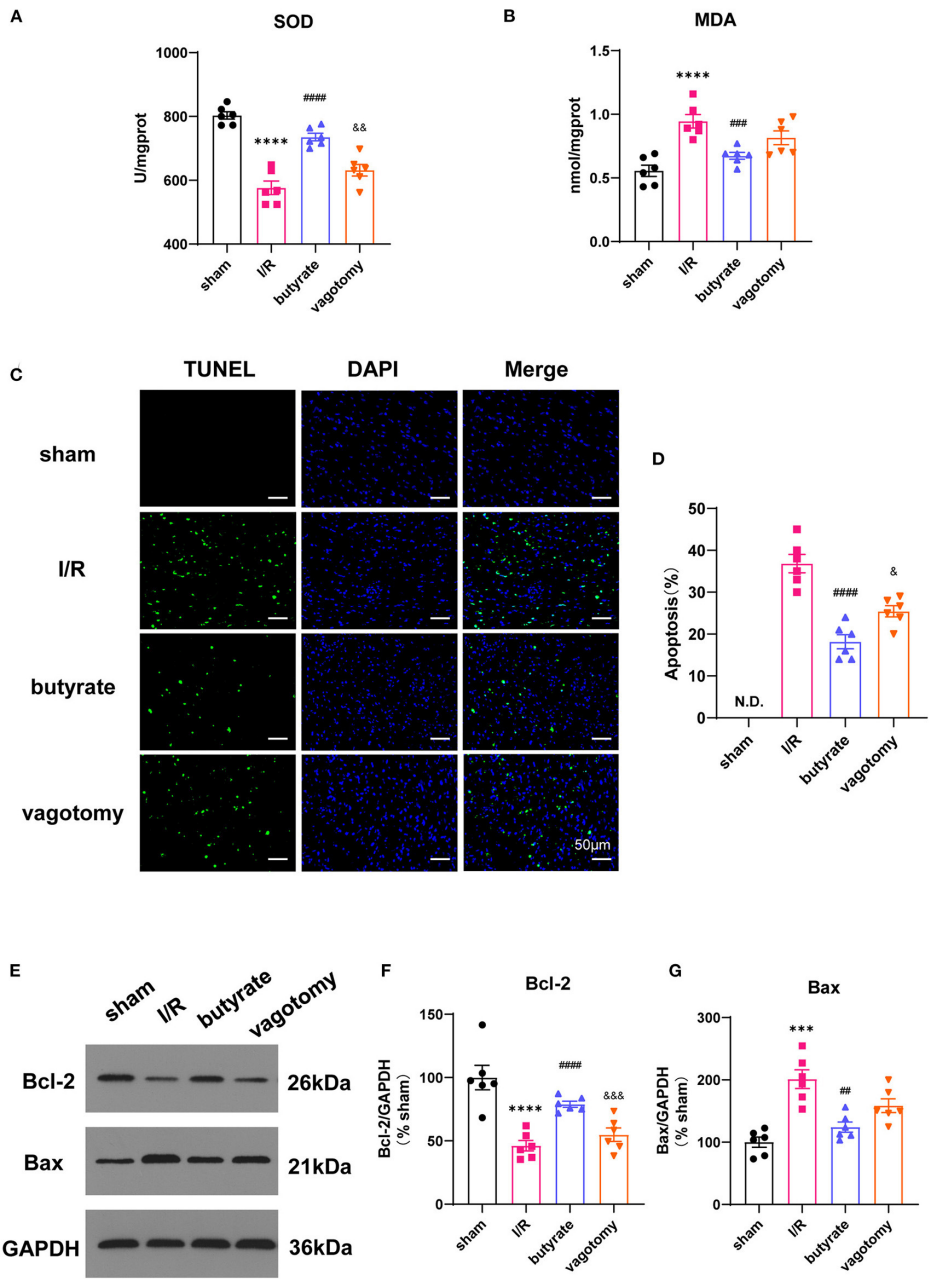
Butyrate treatment improved oxidative stress and cell apoptosis after myocardial I/R, and vagotomy diminished this protective effect. **(A)** Myocardial MDA and **(B)** SOD activities. **(C)** Representative images of TUNEL staining. Myocardial tissues were stained and analyzed with TUNEL (green) and counterstained with DAPI (blue). **(D)** Quantitative analysis of TUNEL positive cells expressed as percentage of DAPI stained cells. N.D., no detectable. Compared group: I/R, butyrate, vagotomy. **(E)** Representative images of Western blot of Bax and Bcl-2 expression from sham, I/R, butyrate, and vagotomy groups. **(F,G)** Relative intensity of Bax/Bcl2 over GAPDH (% sham). ***p < 0.001 vs. sham group, ****p < 0.0001 vs. sham group; ^##^*p* < 0.01 vs. I/R group, ^###^*p* < 0.001 vs. I/R group, ^####^*p* < 0.0001 vs. I/R group; ^&^*p* < 0.05, ^&&^*p* < 0.01, ^&&&^*p* < 0.001 vs. butyrate group. SOD, superoxide dismutase; MDA, malondialdehyde; DAPI, 4',6-diamidino-2-phenylindole; I/R, ischemia/reperfusion; Bcl-2, B-cell lymphoma-2; Bax, BCL2-Associated X; GAPDH, glyceraldehyde-3-phosphate dehydrogenase.

We further investigated the effects on oxidative stress and cell apoptosis. Ischemia/reperfusion significantly increased the levels of SOD and decreased the level of MDA, and these changes were significantly alleviated by butyrate treatment (Figures 2A,B). The number of TUNEL-positive cells in the I/R group was significantly higher than that in the sham, and butyrate treatment decreased apoptosis (Figures 2C,D). The expression of the apoptosis-related proteins Bax and Bcl-2 was also investigated, and the results showed that butyrate significantly decreased Bax expression and increased Bcl-2 expression compared with the levels found in the I/R group (Figures 2E–G). These findings indicate that butyrate treatment leads to a reduction in oxidative stress and cell apoptosis after I/R. In contrast, the vagotomy group presented significantly higher levels of oxidative stress and apoptosis and decreased Bcl-2 protein expression than the butyrate group (Figure 2).

### Butyrate Treatment Inhibited Inflammation in the Heart and Serum After Myocardial I/R, and Vagotomy Diminished This Protective Effect

**Figure 3:**
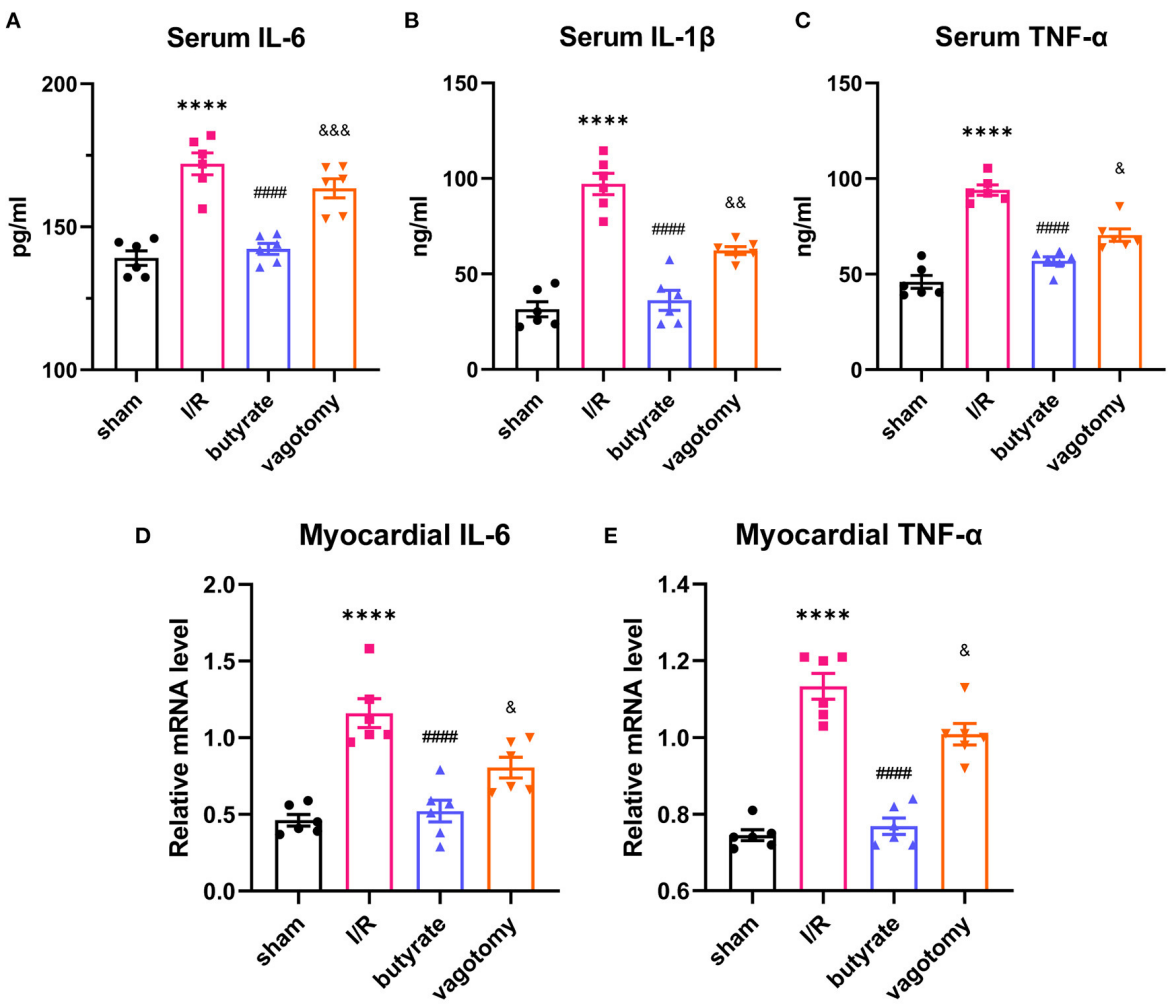
Butyrate treatment inhibited inflammation in the heart and serum after myocardial I/R, and vagotomy diminished this protective effect. **(A)** Serum IL-6, **(B)** serum IL-1β, and **(C)** serum TNF-α levels. **(D)** Myocardial IL-6 and **(E)** TNF-α relative mRNA levels. ****p < 0.0001 vs. sham group; ^####^*p* < 0.0001 vs. I/R group; ^&^*p* < 0.05, ^&&^*p* < 0.01, ^&&&^*p* < 0.001 vs. butyrate group. IL-6, interleukin-6; IL-1β, interleukin-1β; TNF-α, tumor necrosis factor-α; I/R, ischemia/reperfusion.

The serum IL-6, IL-1β, and TNF-α levels and relative mRNA levels of IL-6 and TNF-α were evaluated to measure the inflammation levels. Compared with those in the sham group, the levels of inflammatory factors were elevated in the I/R group, and this effect was attenuated by the administration of butyrate. In contrast, the vagotomy group showed significantly higher levels of serum IL-6, IL-1β, and TNF-α and higher relative mRNA levels of IL-6 and TNF-α than the butyrate group (Figure 3).

### Butyrate Induced Protective Modulation of the Transcriptional Expression Profiles Obtained by I/R, and This Protective Effect Was Reversed by Vagotomy

**Figure 4:**
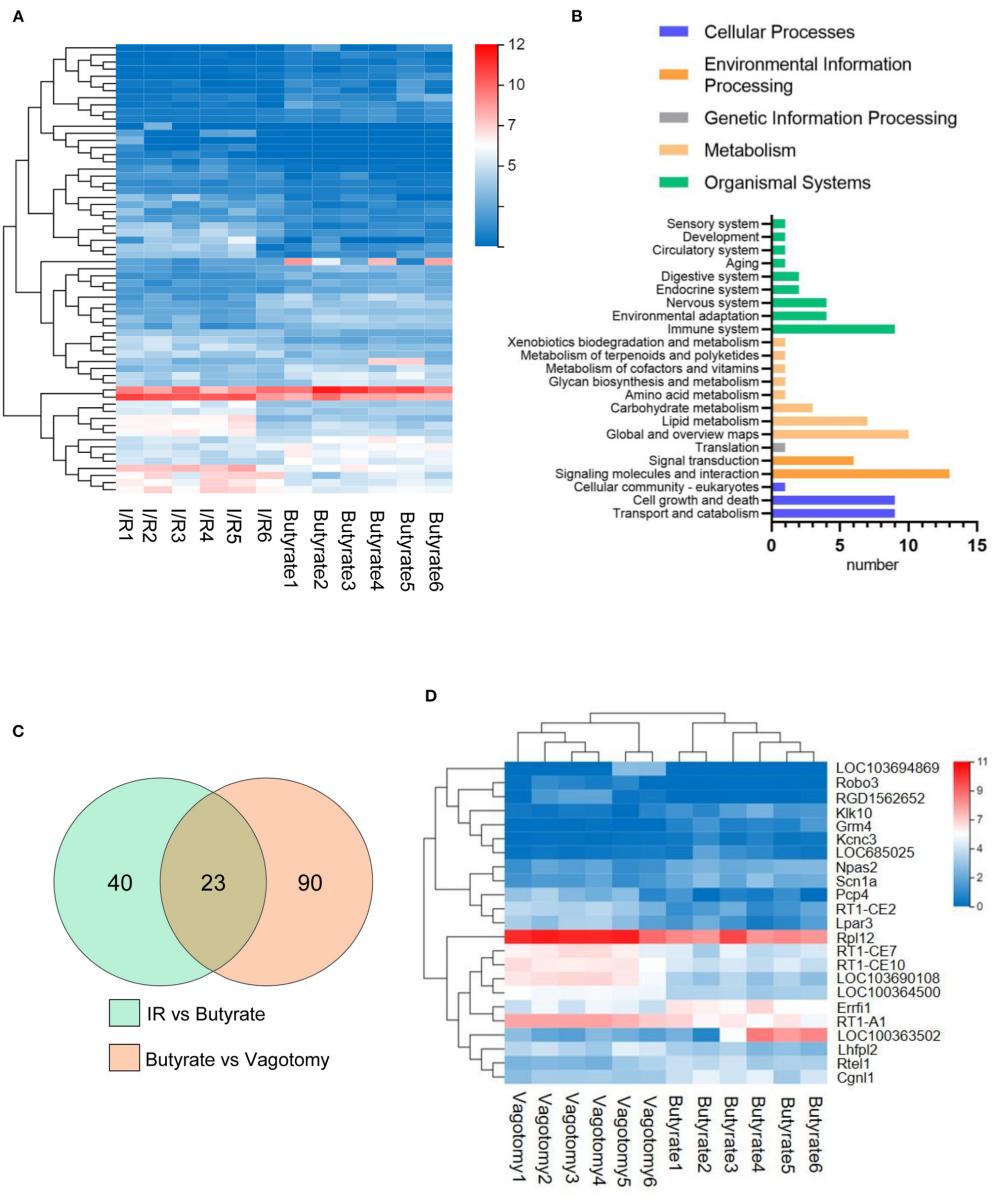
Butyrate induced protective modulation of the transcriptional expression profiles obtained by I/R, and this protective effect was reversed by vagotomy. **(A)** Heat map showing up-regulated or down-regulated DEGs in myocardium induced by butyrate; **(B)** KEGG pathway classification of DEGs changed by butyrate. **(C)** Venn map of DGEs among I/R, butyrate, and vagotomy group. **(D)** Heat map showing expression of butyrate altered genes reversed by vagotomy. DEGs, differentially expressed genes.

To investigate the underlying mechanisms involved in the effects of butyrate treatment and vagotomy, we analyzed the transcriptome profiles of the cardiac tissue of rats belonging to the I/R, butyrate and vagotomy groups by RNA-seq analysis and identified the differentially expressed genes. Thirty and 33 genes were significantly upregulated and downregulated, respectively, in the butyrate group compared with the I/R group (Figure 4A). First, we preliminarily analyzed the KEGG pathway classifications of the differentially expressed genes between the I/R and butyrate groups and found that butyrate could change the expression of genes belonging to “Signaling molecules and interaction,” “Immune system,” “Cell growth and death,” and “Global and overview maps” (Figure 4B). We also performed a GO functional enrichment analysis and found that the butyrate-induced alterations are mainly involved in the immune response, antigen processing, and presentation (Supplementary Figure 2).

**Figure 5:**
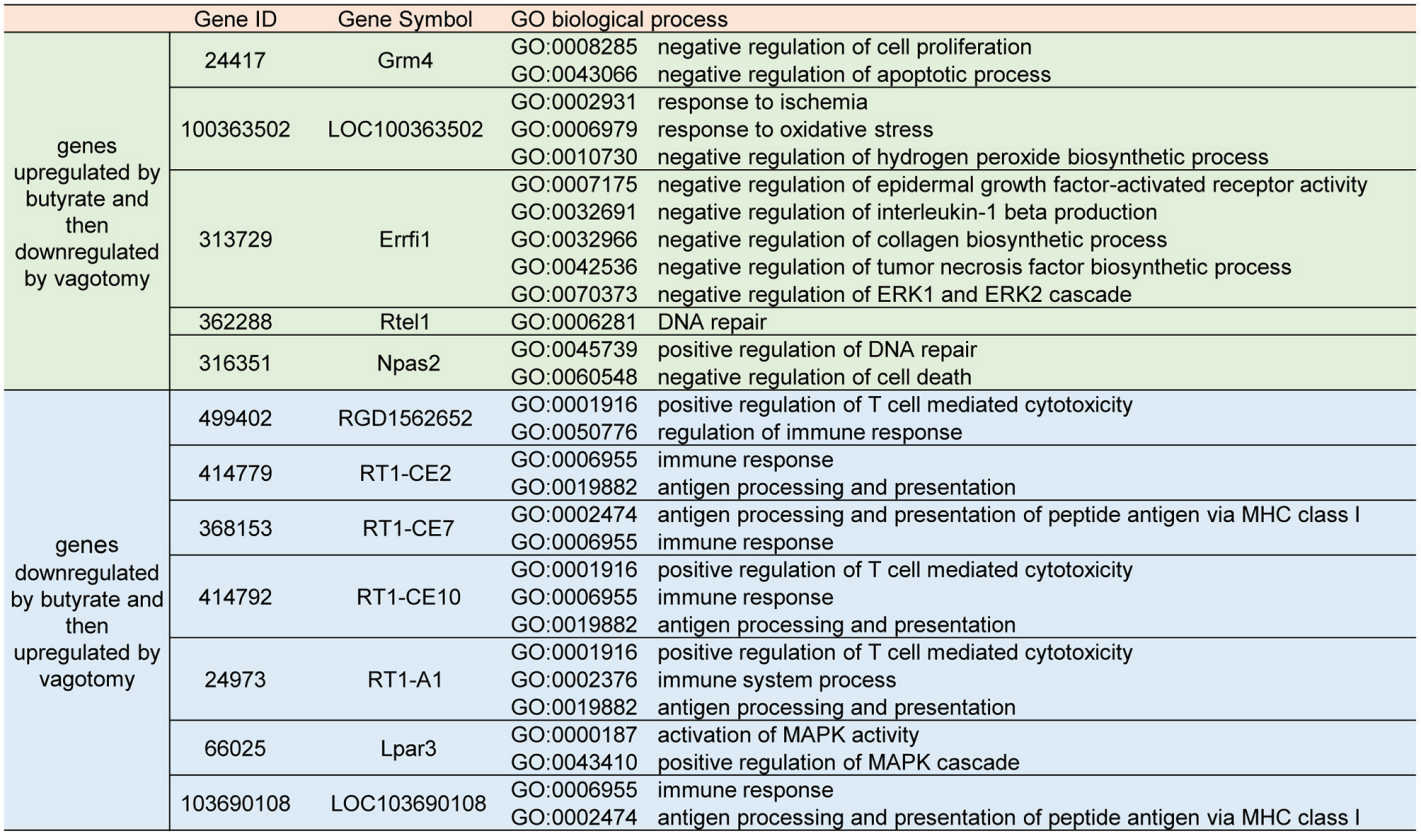
Part of GO biological process annotations of butyrate altered genes reversed by vagotomy.

However, the butyrate treatment-induced changes in gene expression were partly reversed by vagotomy (Figure 4C), and a heat map analysis of the genes was performed (Figure 4D). A total of 10 genes were upregulated by butyrate and then downregulated by vagotomy. According to the GO annotations, these genes are related to negative regulation of apoptotic processes, oxidative stress, and proinflammation. Moreover, 13 genes were downregulated by butyrate and then upregulated by vagotomy. According to the GO annotations, these genes are related to positive regulation of T cell-mediated cytotoxicity and activation of MAPK activity (Figure 5). These findings indicate that vagotomy weakens the protective effect of butyrate treatment.

### Butyrate Treatment Reversed the High Sympathetic Tone After Myocardial I/R, and Vagotomy Diminished This Protective Effect

**Figure 6:**
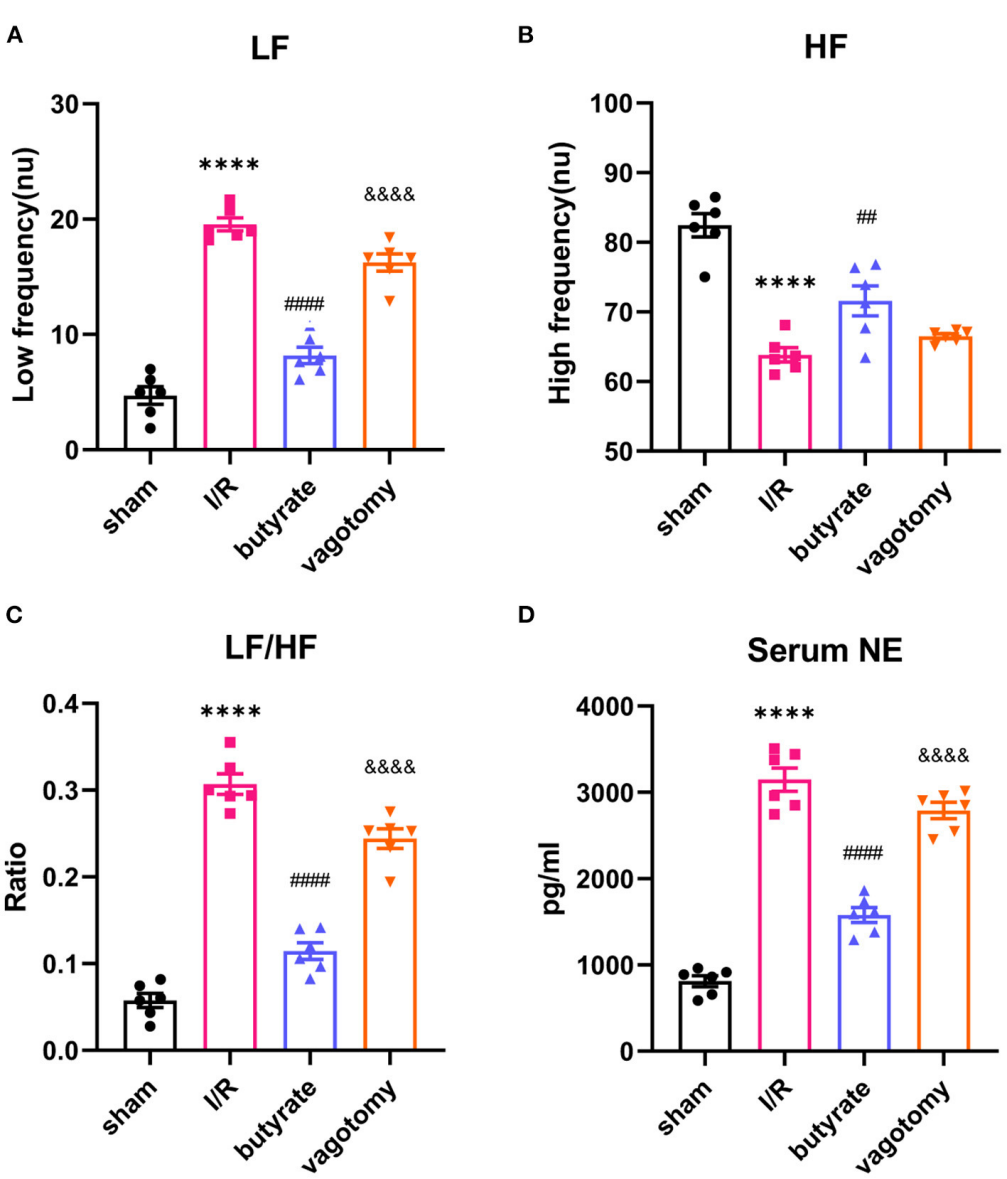
Butyrate treatment reversed the high sympathetic tone after myocardial I/R, and vagotomy diminished this protective effect. **(A)** LF, **(B)** HF, and **(C)** LF/HF. **(D)** Serum NE. ****p < 0.0001 vs. sham group; ^##^*p* < 0.01, ^####^*p* < 0.0001 vs. I/R group; ^&&&&^*p* < 0.0001 vs. butyrate group. LF, low frequency; HF, high frequency; NE, norepinephrine; I/R, ischemia/reperfusion.

For evaluation of the sympathetic and vagal tones, we analyzed the HRV in rats belonging to the different groups. The LF and LF/HF ratio in the I/R group were significantly higher than that in the sham group, but the HF was decreased. However, butyrate treatment reversed these changes induced by I/R (Figures 6A–C). Additionally, the significant increase in the serum NE levels found in the I/R group was reversed in the rats treated with butyrate (Figure 6D). In contrast, the vagotomy group presented significantly higher levels of LF, LF/HF, and NE than the butyrate group (Figure 6). These findings demonstrate that butyrate treatment is able to improve the autonomic balance after myocardial I/R and that the protective effect of butyrate is diminished by vagotomy.

### Butyrate Treatment Suppressed the Central Sympathetic Nervous System and Sympathetic Outflow to the Heart, and Vagotomy Diminished This Protective Effect

**Figure 7:**
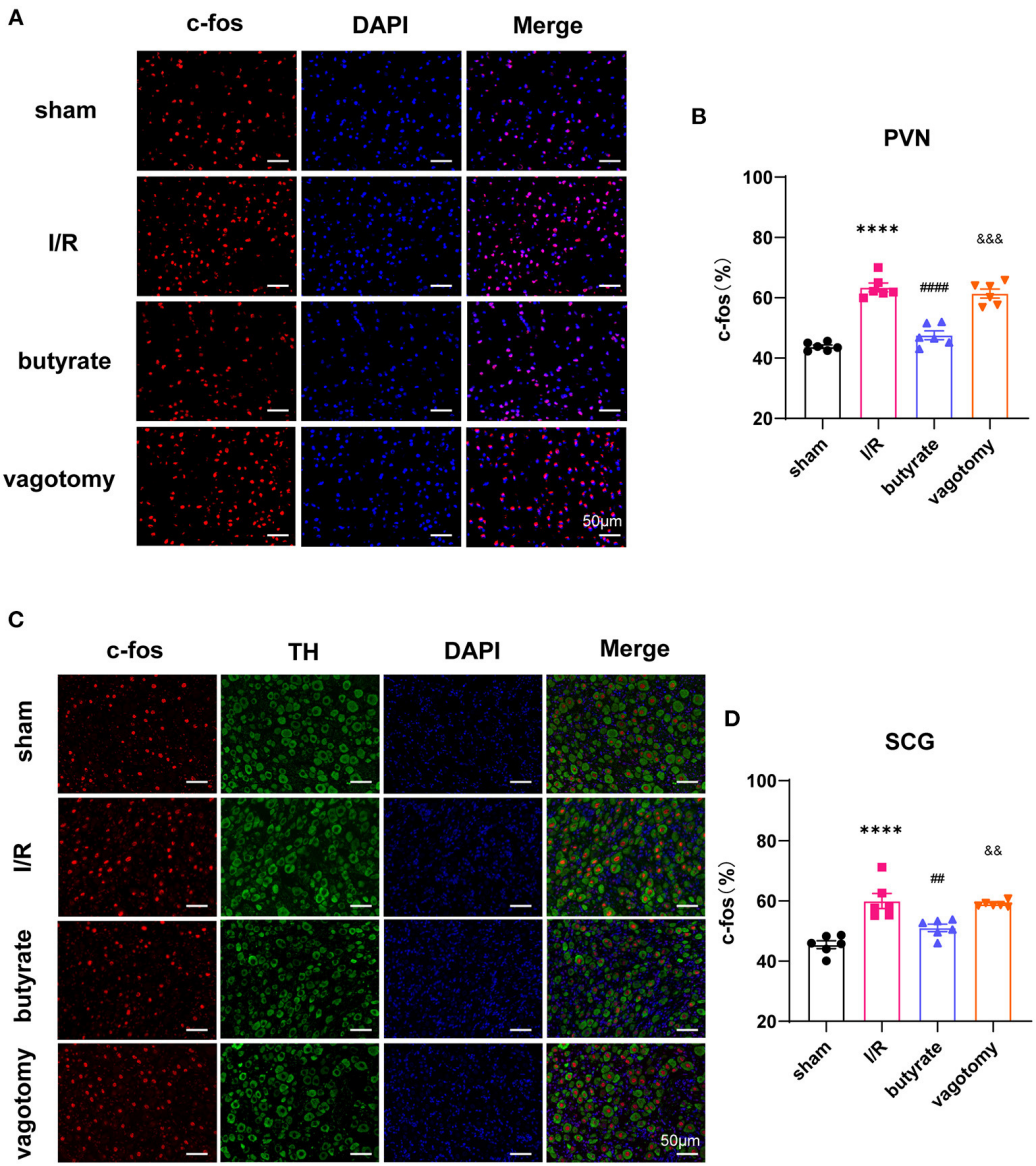
Butyrate treatment suppressed the central sympathetic nervous system and sympathetic outflow to the heart, and vagotomy diminished this protective effect. **(A)** Representative immunofluorescence staining for c-fos in the PVN in all groups. **(B)** Quantitative analysis of c-fos expressed as percentage of DAPI stained cells. **(C)** Representative immunofluorescence staining for c-fos in the SCG in all groups. **(D)** Quantitative analysis of c-fos expressed as percentage of TH+ stained cells. ****p < 0.0001 vs. sham group; ^##^*p* < 0.01, ^####^*p* < 0.0001 vs. I/R group; ^&&^*p* < 0.01, ^&&&^*p* < 0.001 vs. butyrate group. DAPI, 4',6-diamidino-2-phenylindole; I/R, ischemia/reperfusion; PVN, paraventricular nucleus; TH, tyrosine hydroxylase; SCG, superior cervical ganglion.

Anatomical evidence shows that neural pathways from the gut to the PVN are biologically plausible. We investigated whether butyrate can alter the neural activity of the PVN. The cFos expression level was used to evaluate neuronal activation. The results showed that cFos expression within the PVN was markedly downregulated in the butyrate group compared with that in the I/R group (Figures 7A,B). The SCG is a major trunk of the sympathetic pathway (20), and inhibition of the SCG could significantly suppress sympathetic activity contributing to cardiac protection (21). The results showed that cFos expression within the SCG in the butyrate group was also suppressed compared with that in the I/R group, which indicated that butyrate could inhibit the central sympathetic nervous system and sympathetic outflow to the heart (Figures 7C,D). However, vagotomy abolished the effects of butyrate on the PVN and SCG (Figure 7). These results indicate that the protective effect of butyrate on the autonomic imbalance might be due to the gut-brain circuit.

## Discussion

**Figure 8:**
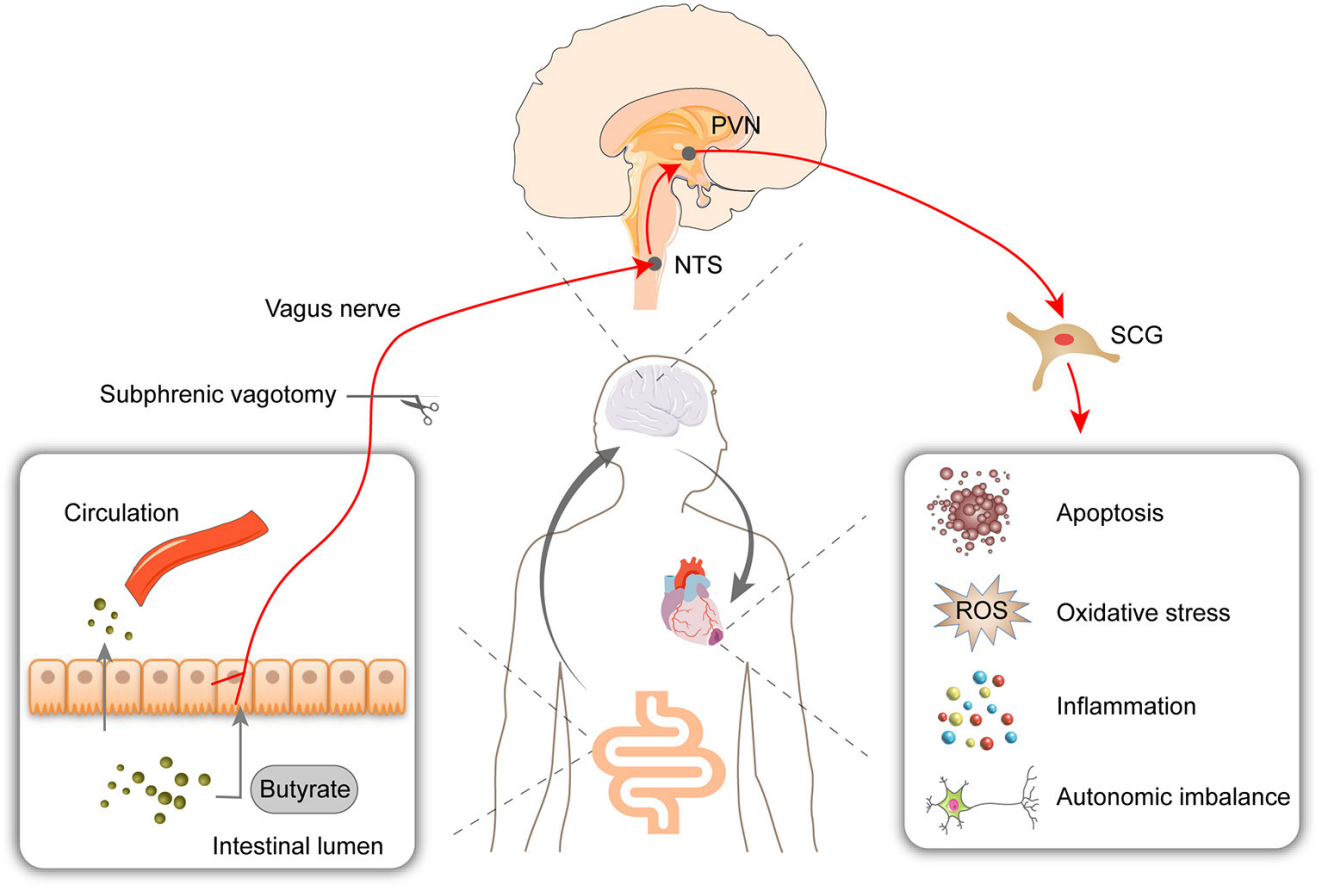
Oral supplementation with butyrate attenuated myocardial ischemia/reperfusion injury via a gut-brain neural circuit, and vagotomy diminished this effect. Other than entering circulation, butyrate might also act on the vagus nerve. The protective effect on autonomic imbalance, oxidative stress, cell apoptosis, and inflammation might due to improving the activation of PVN and SCG induced by I/R. PVN, paraventricular nucleus; NTS, nucleus tractus solitarius; SCG, superior cervical ganglion; ROS, reactive oxygen species.

This study showed that butyrate exerts a protective effect on myocardial I/R injury, as evidenced by reductions in the infarct size, oxidative stress, cell apoptosis, and inflammation. The RNA-Seq results showed that the butyrate-induced protective changes in gene expression were partly reversed by vagotomy. Moreover, we identified a “gut-brain” circuit neural mechanism through which butyrate can modulate the sympathetic outflow from brain to heart and improve the autonomic imbalance (Figure 8).

Previous findings have shown that depletion of the gut microbiota causes detrimental effects and that SCFA supplementation exerts multiple beneficial effects on MI, hypertension, and other diseases (7, 8). In this study, we demonstrated that butyrate could improve myocardial I/R by reducing apoptosis, oxidative stress, and inflammation. Consistent with our results, other studies have shown that the administration of butyrate or the stimulation of intestinal butyrate production via probiotics improves inflammatory markers and oxidative stress in various disease models, including obesity, diabetes, lung ischemia-reperfusion injury, and ischemic stroke (22–25). Most of the mechanisms are involved in the direct activation of GPRs or the inhibition of HDACs. Butyrate can induce intracellular signaling pathways by interacting with GPRs on target cells and acts as a HDAC inhibitor.

However, in addition to its action on cardiomyocytes, evidence has shown that oral supplementation with butyrate might also function through gut-brain neural mechanisms that depend on afferent vagus nerve signaling. A previous study showed that butyrate could improve energy metabolism by the gut-brain neural circuit and that subdiaphragmatic vagotomy abolishes these effects (9). The administration of butyrate to the colon decreases both the heart rate and blood pressure, and subdiaphragmatic vagotomy significantly reduces this hypotensive effect (10). In addition, it has been shown that microbiota and SCFAs can modulate sympathetic neurons and that this effects mainly rely on vagal afferents (11). Based on these findings, we hypothesized that butyrate could affect heart health via a neural mechanism. To verify this conjecture, we performed preliminarily experiments using subdiaphragmatic vagotomized animals. We found that the protective effect of butyrate was diminished by vagotomy, which indicates that the vagus nerve mediates the protective function of butyrate. Moreover, the changes in the transcriptome of the heart were investigated by RNA-Seq. The results showed that the butyrate-induced protective changes in gene expression are partly reversed by vagotomy and are mainly involved in the regulation of the immune response, apoptotic process, oxidative stress, and proinflammation.

Cardiovascular diseases are accompanied by autonomic nervous system imbalance. The development and progression of myocardial I/R are caused by multiple factors and inextricably linked to autonomic imbalance (26, 27). Some researchers have shown that butyrate could improve cardiac function and sympathetic neural remodeling following myocardial infarction in a rat model (28). Butyrate may stimulate the sensory fibers of the vagus nerve that project to the brain centers controlling the circulatory system. This stimulation results in decreased tonic sympathetic activity and produces a decrease in the arterial blood pressure (10). Moreover, another study revealed that microbiota depletion leads to elevated cFos in the lateral paragigantocellular nucleus/rostral ventrolateral medulla (LPGi/RVLM) and coeliac-superior mesenteric ganglia (CG-SMG) through the vagal afferent pathway, and the administration of butyrate is sufficient to suppress cFos expression in the gut sympathetic ganglia (11). The increased cardiac sympathetic tone in Ang II-treated mice is ameliorated by butyrate (29). In our study, we found that butyrate could reverse myocardial I/R-induced autonomic imbalance and these effects could be abolished by vagotomy.

Based on these results, we further hypothesized that butyrate might suppress the central sympathetic nervous system through the vagal afferent pathway. Evidence has shown that AMI induces sympathetic nerve hyperactivity and that targeting the PVN may be an approach to ameliorate sympathetic overexcitation and could induce a cardioprotective effect (30). In addition, high levels of NE might cause oxidative stress and induce cell apoptosis (31, 32). The SCG is a major trunk of the sympathetic pathway (20), and inhibition of the SCG could significantly suppress sympathetic activity contributing to cardiac protection (21). A significant body of evidence demonstrates that the nucleus tractus solitarius (NTS) can directly integrate gut sensory information from vagal sensory neurons and that the nuclei connect to the PVN (33, 34). Therefore, we detected the neuronal activity of the PVN and SCG and found that butyrate significantly suppresses the PVN and SCG, as verified by decreased cFos expression. However, the effects on neuronal activity were abolished by vagotomy, which suggests that the protective effects of butyrate on myocardial I/R through a gut-brain neural pathway might be due to reduced sympathetic outflow from the brain to the heart.

Although progress has been made in the prevention and therapy of myocardial I/R injury, the efficacy of the current treatments is still limited. Our study provides new insights into the cardioprotective effects of butyrate, and our data suggest that dietary supplementation with fiber or the application of butyrate may have therapeutic potential for myocardial I/R injury.

## Study Limitations

As a preliminary study to investigate the neural mechanism of butyrate treatment, we only demonstrated the key role of the vagus nerve and drew the speculative conclusion that a reduction in sympathetic outflow from the brain might contribute to the protective effect of butyrate. It is shown that butyrate could act on extrinsic enteric–associated neurons (eEAN), comprised of sensory afferents and autonomic efferents, thereby affecting the signal transmission from the vagus nerve to the brain, likely mediated by GPR41 (11). Furthermore, butyrate administered into the colon produced a significant hypotensive effect which was diminished by the vagotomy and intracolonic pretreatment with a non-specific antagonist of GPR41/43. These findings also suggested that the hypotensive response involves vagus nerve and GPR41/43 (10). Further elucidation of the actions of butyrate in the gut and brain requires further exploration, and additional information may provide a better understanding of this gut-brain axis. Moreover, we only performed an oral chronic experiment, and whether acute supplementation exerts a similar effect requires further experiments, which will provide more in-depth insights and thereby a more comprehensive understanding of the effects of butyrate on myocardial I/R.

## Conclusions

Butyrate treatments significantly improve myocardial I/R injury via a gut-brain neural circuit, and the cardioprotective effect of butyrate is likely mediated by the “PVN-SCG” sympathetic pathway.

## Data Availability Statement

The datasets presented in this study can be found in online repositories. The names of the repository/repositories and accession number(s) can be found at: Sequence Read Archive, PRJNA741778.

## Ethics Statement

The animal study was reviewed and approved by the Animal Welfare and Ethics Committee of Renmin Hospital of Wuhan University.

## Author Contributions

ZY, JH, and HC designed the study and wrote the manuscript. YW, LZ, MW, RZ, and XJ collected laboratory data. GZ, CW, TX, MX, and XW performed the statistical analysis. XZ and HJ edited manuscript. All authors contributed to the article and approved the submitted version.

## Funding

This work was supported by the National Key R&D Program of China (2017YFC1307802) and the National Natural Science Foundation of China (81970287, 81530011, and 81770364).

## Conflict of Interest

The authors declare that the research was conducted in the absence of any commercial or financial relationships that could be construed as a potential conflict of interest.

## Publisher's Note

All claims expressed in this article are solely those of the authors and do not necessarily represent those of their affiliated organizations, or those of the publisher, the editors and the reviewers. Any product that may be evaluated in this article, or claim that may be made by its manufacturer, is not guaranteed or endorsed by the publisher.

## References

[1] Yellon DM Hausenloy DJ. Myocardial reperfusion injury. N Engl J Med. (2007) 357:1121–35. 10.1056/NEJMra071667 17855673

[2] Gottlieb RA Engler RL. Apoptosis in myocardial ischemia-reperfusion. Ann N Y Acad Sci. (1999) 874:12–26. 10.1111/j.1749-6632.1999.tb09255.x 10415551

[3] Frangoginis NG Smith CW Entman ML. The inflammatory response in myocardial infarction. Cardiovasc Res. (2002) 53:31–47. 10.1016/s0008-6363(01)00434-5 11744011

[4] Cummings JH Pomare EW Branch WJ Naylor CP Macfarlane GT. Short chain fatty acids in human large intestine, portal, hepatic and venous blood. Gut. (1987) 28:1221–7. 10.1136/gut.28.10.1221 3678950 PMC1433442

[5] Tang TWH Chen HC Chen CY Yen CYT Lin CJ Prajnamitra RP et al. Loss of gut microbiota alters immune system composition and cripples postinfarction cardiac repair. Circulation. (2019)139:647–59. 10.1161/CIRCULATIONAHA.118.035235 30586712

[6] Zhai X Lin D Zhao Y Li W Yang X. Effects of dietary fiber supplementation on fatty acid metabolism and intestinal microbiota diversity in C57BL/6J mice fed with a high-fat diet. J Agric Food Chem. (2018) 66:12706–18. 10.1021/acs.jafc.8b05036 30411889

[7] Song T Guan X Wang X Qu S Zhang S Hui W. Dynamic modulation of gut microbiota improves post-myocardial infarct tissue repair in rats via butyric acid-mediated histone deacetylase inhibition. FASEB J. (2021) 35:e21385. 10.1096/fj.201903129RRR 33565193

[8] Zhang L Deng M Lu A Chen Y Chen Y Wu C. Sodium butyrate attenuates angiotensin II-induced cardiac hypertrophy by inhibiting COX2/PGE2 pathway via a HDAC5/HDAC6-dependent mechanism. J Cell Mol Med. (2019) 23:8139–50. 10.1111/jcmm.14684 31565858 PMC6850921

[9] Li Z Yi CX Katiraei S Kooijman S Zhou E Chung CK. Butyrate reduces appetite and activates brown adipose tissue via the gut-brain neural circuit. Gut. (2018) 67:1269–79. 10.1136/gutjnl-2017-314050 29101261

[10] Onyszkiewicz M Gawrys-Kopczynska M Konopelski P Aleksandrowicz M Sawicka A Kozniewska E. Butyric acid, a gut bacteria metabolite, lowers arterial blood pressure via colon-vagus nerve signaling and GPR41/43 receptors. Pflugers Arch. (2019) 471:1441–53. 10.1007/s00424-019-02322-y 31728701 PMC6882756

[11] Muller PA Schneeberger M Matheis F Wang P Kerner Z Ilanges A. Microbiota modulate sympathetic neurons via a gut-brain circuit. Nature. (2020) 583:441–6. 10.1038/s41586-020-2474-7 32641826 PMC7367767

[12] Yu CD Xu QJ Chang RB. Vagal sensory neurons and gut-brain signaling. Curr Opin Neurobiol. (2020) 62:133–40. 10.1016/j.conb.2020.03.006 32380360 PMC7560965

[13] Huang B Yu L Scherlag BJ Wang S He B Yang K. Left renal nerves stimulation facilitates ischemia-induced ventricular arrhythmia by increasing nerve activity of left stellate ganglion. J Cardiovasc Electrophysiol. (2014) 25:1249–56. 10.1111/jce.12498 25066536

[14] Wang S Zhou X Huang B Wang Z Liao K Saren G. Spinal cord stimulation protects against ventricular arrhythmias by suppressing left stellate ganglion neural activity in an acute myocardial infarction canine model. Heart Rhythm. (2015) 12:1628–35. 10.1016/j.hrthm.2015.03.023 25778432

[15] Pyner S. The paraventricular nucleus and heart failure. Exp Physiol. (2014) 99:332–9. 10.1113/expphysiol.2013.072678 24317407

[16] Santisteban MM Qi Y Zubcevic J Kim S Yang T Shenoy V. Hypertension-linked pathophysiological alterations in the gut. Circ Res. (2017) 120:312–23. 10.1161/CIRCRESAHA.116.309006 27799253 PMC5250568

[17] Wieczorek M Swiergiel AH Pournajafi-Nazarloo H Dunn AJ. Physiological and behavioral responses to interleukin-1beta and LPS in vagotomized mice. Physiol Behav. (2005) 85:500–11. 10.1016/j.physbeh.2005.05.012 15996692 PMC2293826

[18] Polhemus DJ Gao J Scarborough AL Trivedi R McDonough KH Goodchild TT. Radiofrequency renal denervation protects the ischemic heart via inhibition of GRK2 and increased nitric oxide signaling. Circ Res. (2016) 119:470–80. 10.1161/CIRCRESAHA.115.308278 27296507 PMC4959827

[19] Dias da Silva VJ Tobaldini E Rocchetti M Wu MA Malfatto G Montano N. Modulation of sympathetic activity and heart rate variability by ivabradine. Cardiovasc Res. (2015) 108:31–8. 10.1093/cvr/cvv180 26101263

[20] Wang FB Cheng PM Chi HC Kao CK Liao YH. Axons of passage and inputs to superior cervical ganglion in rat. Anat Rec (Hoboken). (2018) 301:1906–16. 10.1002/ar.23953 30338669

[21] Shi Y Li Y Yin J Hu H Xue M Li X. A novel sympathetic neuronal GABAergic signalling system regulates NE release to prevent ventricular arrhythmias after acute myocardial infarction. Acta Physiol (Oxf). (2019) 227:e13315. 10.1111/apha.13315 31116911 PMC6813916

[22] Xu YH Gao CL Guo HL Zhang WQ Huang W Tang SS. Sodium butyrate supplementation ameliorates diabetic inflammation in db/db mice. J Endocrinol. (2018) 238:231–44. 10.1530/JOE-18-0137 29941502

[23] Aguilar EC da Silva JF Navia-Pelaez JM Leonel AJ Lopes LG Menezes-Garcia Z. Sodium butyrate modulates adipocyte expansion, adipogenesis, and insulin receptor signaling by upregulation of PPAR-γ in obese Apo E knockout mice. Nutrition. (2018) 47:75–82. 10.1016/j.nut.2017.10.007 29429540

[24] Ying XD Wei G An H. Sodium butyrate relieves lung ischemia-reperfusion injury by inhibiting NF-κB and JAK2/STAT3 signaling pathways. Eur Rev Med Pharmacol Sci. (2021) 25:413–22. 10.26355/eurrev_202101_24409 33506931

[25] Park MJ Sohrabji F. The histone deacetylase inhibitor, sodium butyrate, exhibits neuroprotective effects for ischemic stroke in middle-aged female rats. J Neuroinflammation. (2016) 13:300. 10.1186/s12974-016-0765-6 27905989 PMC5131416

[26] Frangogiannis NG. The inflammatory response in myocardial injury, repair, and remodelling. Nat Rev Cardiol. (2014) 11:255–65. 10.1038/nrcardio.2014.28 24663091 PMC4407144

[27] Hartupee J Mann DL. Neurohormonal activation in heart failure with reduced ejection fraction. Nat Rev Cardiol. (2017) 14:30–8. 10.1038/nrcardio.2016.163 27708278 PMC5286912

[28] Jiang X Huang X Tong Y Gao H. Butyrate improves cardiac function and sympathetic neural remodeling following myocardial infarction in rats. Can J Physiol Pharmacol. (2020) 98:391–9. 10.1139/cjpp-2019-0531 31999473

[29] Kim S Goel R Kumar A Qi Y Lobaton G Hosaka K. Imbalance of gut microbiome and intestinal epithelial barrier dysfunction in patients with high blood pressure. Clin Sci (Lond). (2018) 132:701–18. 10.1042/CS20180087 29507058 PMC5955695

[30] Wang Y Hu H Yin J Shi Y Tan J Zheng L. TLR4 participates in sympathetic hyperactivity Post-MI in the PVN by regulating NF-κB pathway and ROS production. Redox Biol. (2019) 24:101186. 10.1016/j.redox.2019.101186 30978539 PMC6460304

[31] Schraml E Quan P Stelzer I Fuchs R Skalicky M Viidik A. Norepinephrine treatment and aging lead to systemic and intracellular oxidative stress in rats. Exp Gerontol. (2007) 42:1072–8. 10.1016/j.exger.2007.08.003 17851010

[32] de Lima-Seolin BG Nemec-Bakk A Forsyth H Kirk S da Rosa Araujo AS Schenkel PC. Modulates cardiac remodeling by attenuating oxidative stress in H9c2 cardiac cells exposed to norepinephrine. Oxid Med Cell Longev. (2019) 2019:6325424. 10.1155/2019/6325424 31360296 PMC6652037

[33] Gao J Zhang F Sun HJ Liu TY Ding L Kang YM. Transneuronal tracing of central autonomic regions involved in cardiac sympathetic afferent reflex in rats. J Neurol Sci. (2014) 342:45–51. 10.1016/j.jns.2014.04.023 24819915

[34] Han W Tellez LA Perkins MH Perez IO Qu T Ferreira J. A Neural circuit for gut-induced reward. Cell. (2018) 175:665.e23–78.e23. 10.1016/j.cell.2018.08.049 30245012 PMC6195474

