# Supplementary material for: Oral Supplementation With Butyrate Improves Myocardial Ischemia/Reperfusion Injury via a Gut-Brain Neural Circuit
Source: Front Cardiovasc Med. 2021 Sep 23;8:718674. doi: 10.3389/fcvm.2021.718674 (PMC8495014; doi:10.3389/fcvm.2021.718674)
Supplement: Supplementary file 1 [file Data_Sheet_1.PDF]

Supplementary Material

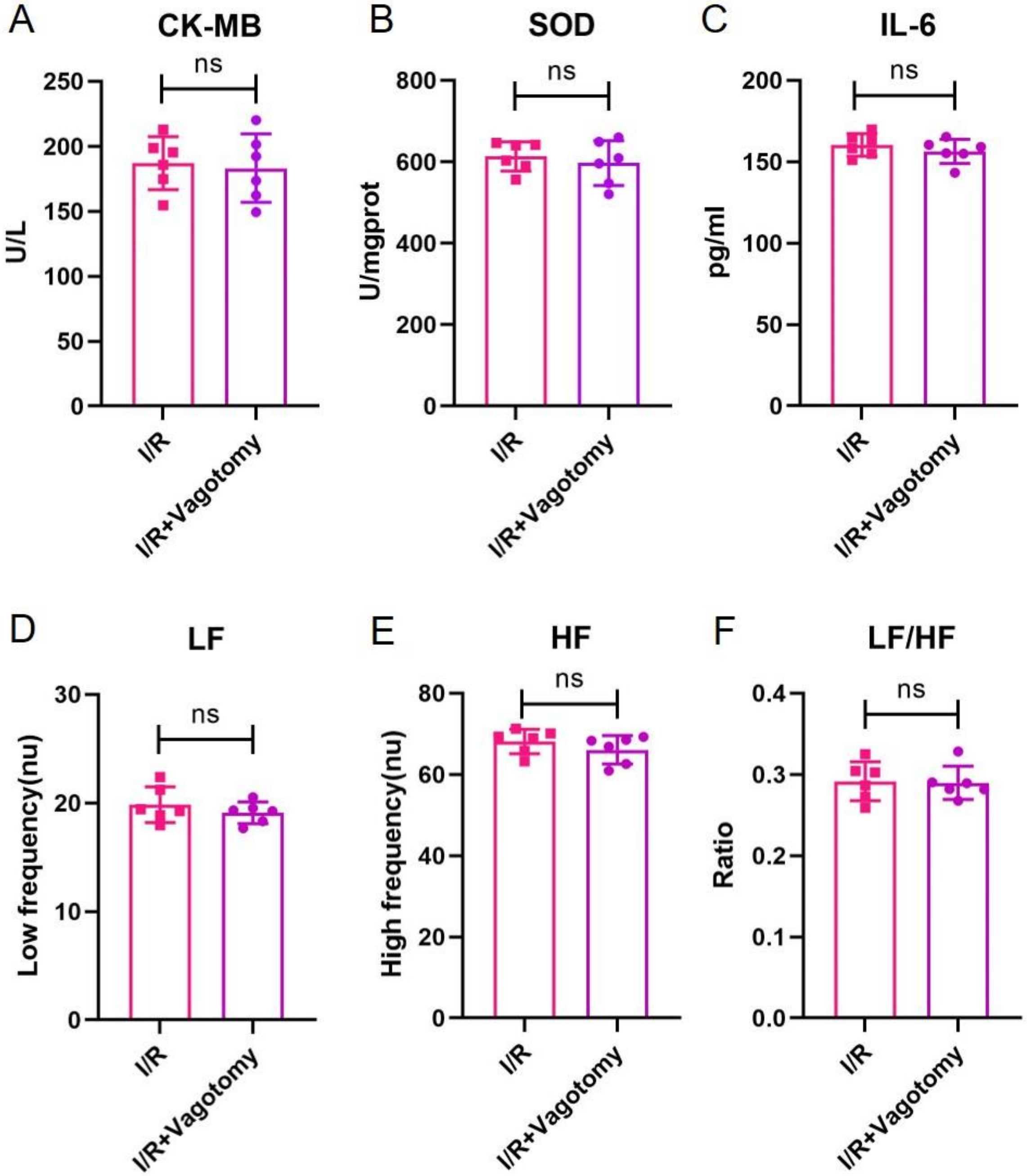

Supplementary Figure 1. Vagotomy had no significant effect on myocardial I/R. (A) Serum CK-MB, (B) SOD activities, (C) serum IL-6. (D) LF, (E) HF and (F) LF/HF. ns: not significant. CK-MB, creatine kinase-MB; SOD, superoxide dismutase; IL-6, interleukin-6; LF, low frequency; HF, high frequency; I/R, ischemia/reperfusion. The data conformed to a normal distribution and was expressed as mean ± standard deviation (SD), and the independent-sample t-test was used for analysis.

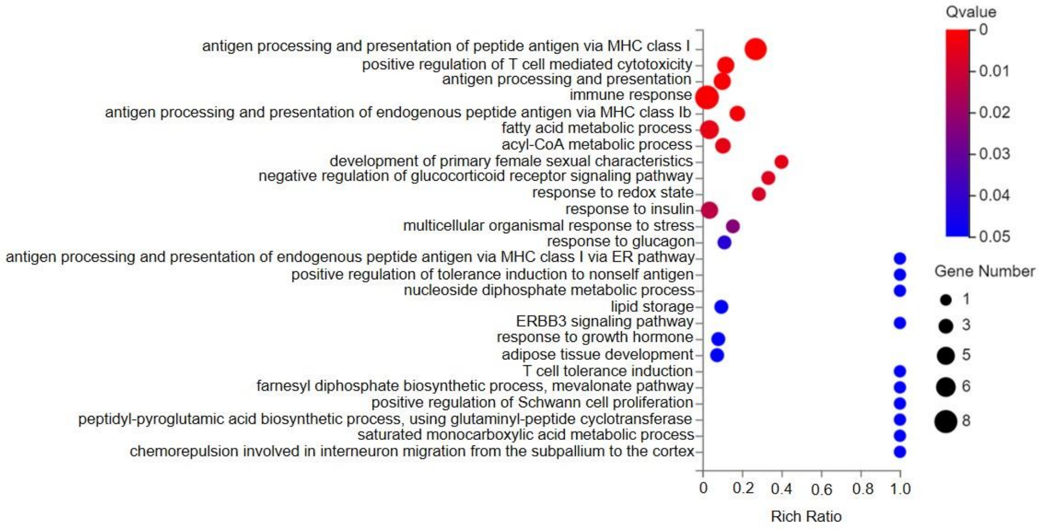

Supplementary Figure 2. Enrichment of GO Biological Process for butyrate altered genes.
